# Comparative outcomes of uncemented and cemented stem revision in managing periprosthetic femoral fractures: a retrospective cohort study

**DOI:** 10.1186/s10195-024-00777-z

**Published:** 2024-07-18

**Authors:** Michael Axenhus, Sebastian Mukka, Martin Magnéli, Olof Sköldenberg

**Affiliations:** 1 Department of Clinical Sciences at Danderyd Hospital, Division of Orthopaedics, Karolinska Institutet, Stockholm, Sweden; 2 Department of Diagnostics and Intervention (Orthopaedics), Umeå University, Umeå, Sweden

**Keywords:** Arthroplasty, Femoral fractures, Revision surgery, Stem fixation, Vancouver classification

## Abstract

**Introduction:**

Periprosthetic femoral fractures (PFFs) following hip arthroplasty, especially Vancouver B2 and B3 fractures, present a challenge due to the association with a loose femoral stem, necessitating either open reduction and internal fixation or stem revision. This study aims to compare outcomes between uncemented and cemented stem revisions in managing Vancouver B2 and B3 fractures, considering factors such as hip-related complications, reoperations, and clinical outcome.

**Methods:**

A retrospective cohort study was conducted at Danderyd Hospital, Sweden, from 2008 to 2022, encompassing operatively treated Vancouver B2 and B3 fractures. Patients were categorized into uncemented and cemented stem revision groups, with data collected on complications, revision surgeries, fracture healing times, and clinical outcomes.

**Results:**

A total of 241 patients were identified. Significant differences were observed between the two groups in patient demographics, with the cemented group comprising older patients and more females. Follow up ranged from 1 to 15 years. Average follow up time was 3.9 years for the cemented group and 5.5 years for the uncemented group. The cemented stems demonstrated lower rates of dislocation (8.9% versus 22.5%, *P* = 0.004) and stem loosening (0.6% versus 9.3%, *P* = 0.004) than the uncemented method. Moreover, the cemented group exhibited shorter fracture healing times (11.4 weeks versus 16.7 weeks, *P* = 0.034). There was no difference in clinical outcome between groups. Mortality was higher in the cemented group.

**Conclusions:**

This retrospective study indicates that cemented stem revision for Vancouver B2–3 fractures is correlated with lower dislocation and stem loosening rates, necessitating fewer reoperations and shorter fracture healing times compared with the uncemented approach. The cemented group had a notably higher mortality rate, urging caution in its clinical interpretation.

*Level of evidence* III

## Introduction

Periprosthetic femoral fractures (PFFs) following hip arthroplasty present a significant challenge in orthopedics and are becoming increasingly common due to the number of primary and revision total hip arthroplasties being performed [1, 2]. The Vancouver classification system categorizes periprosthetic fractures around hip implants into four types (A, B, and C) based on fracture location and extent relative to the implant, aiding surgeons in treatment decisions [3]. Vancouver B2–3 fractures pose a distinct challenge, as they are associated with a loose femoral stem, indicating inherent instability that frequently requires more than fracture stabilization alone [4]. Addressing these fractures involves choosing between open reduction and internal fixation (ORIF) or stem revision.

The management of Vancouver B2–3 fractures often leaned toward stem revision, aiming to restore biomechanical stability, which can be performed using uncemented or cemented techniques. Cemented stem revision could entail higher risks of systemic complications, including bone cement implantation syndrome [5, 6]. On the other hand, uncemented stem revision requires a prosthetic stem designed to allow bone growth for long-term fixation [7]. While this method might reduce the risk of cement-related complications, achieving immediate stability can be challenging, potentially leading to increased risks of fracture displacement or subsidence [8]. The comparative effectiveness of uncemented versus cemented stem revision in treating PFFs is inadequately explored, and management paradigms are changing [9, 10]. The potential risks associated with stem revision necessitate a comprehensive evaluation of the treatment outcomes associated with both methods [11]. This study aims to evaluate clinical outcomes, including mortality, between uncemented and cemented stem revision in the treatment of Vancouver B2 and B3 PFF.

## Methods

### Study design and setting

This retrospective cohort study was conducted at the Orthopedic Department of Danderyd Hospital in Stockholm, Sweden, spanning from 2008 to 2022. Danderyd Hospital, affiliated with the Karolinska Institute, serves approximately 800,000 inhabitants. Data were collected using REDCap electronic data capture tools until September 2023, with a minimum 1-year follow-up postsurgery [12]. Ethical approval was obtained, and the study adhered to the Strengthening the Reporting of Observational Studies in Epidemiology (STROBE) guidelines for reporting observational cohorts [13].

### Participants

Patients were identified from the local surgical planning system, medical records, and the Swedish Arthroplasty register. The study encompassed a consecutive series of all surgically treated periprosthetic Vancouver B2–3 fractures.

### Patient flow and baseline data

**Fig. 1:**
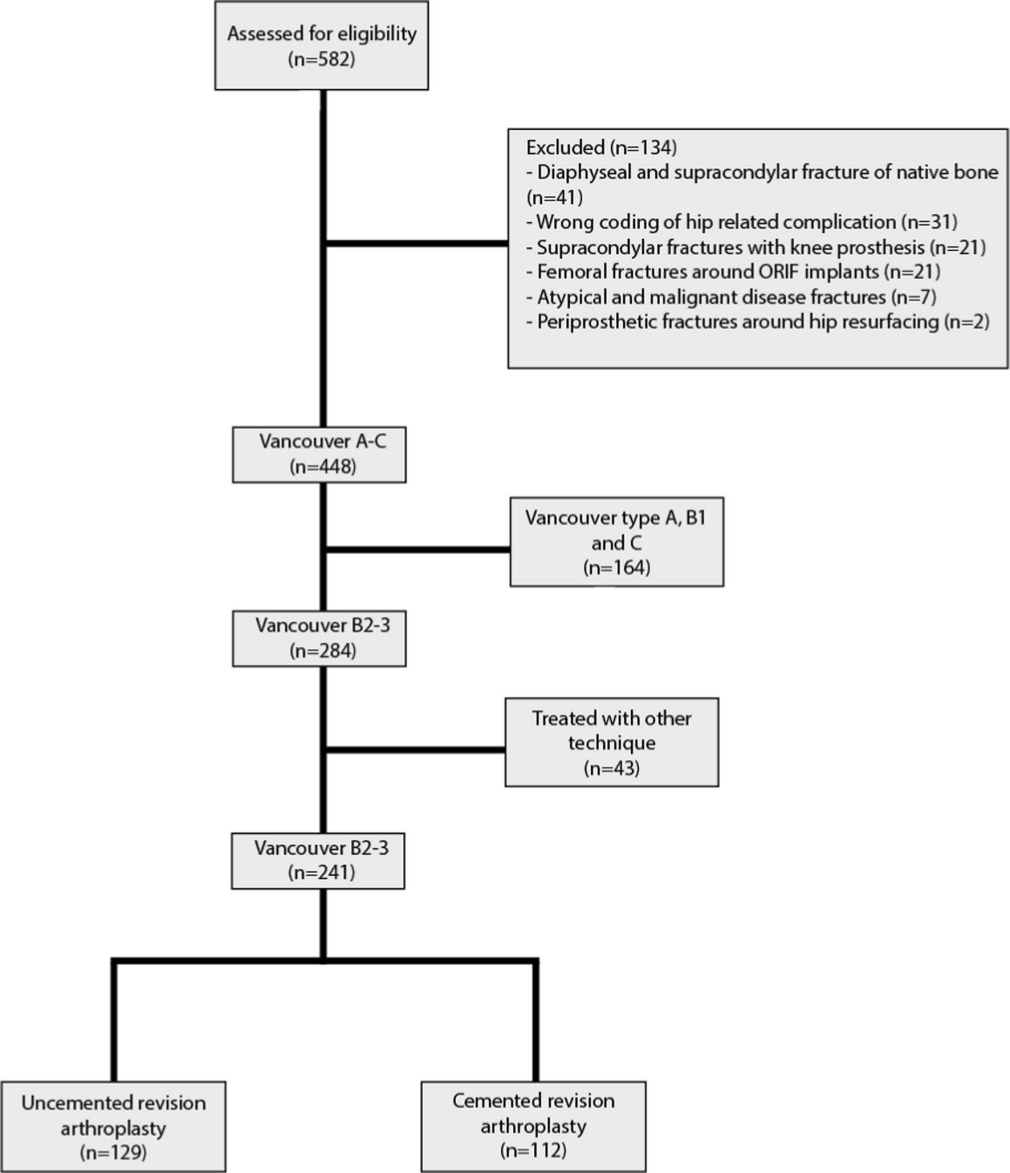
Chart of patient flow

A total of 241 patients were identified during the study period (Fig. 1).

The cemented group was older, had fewer females, and were less cognitive intact. There was no difference in Vancouver class (Table 1).

**Table 1:** Baseline characteristics of study participants

Baseline	Cemented (*n* = 112)	Uncemented (*n* = 129)	*P* value
Age at revision [mean, standard deviation (SD)]	83 ± 7.3 (61–98)	76 ± 9.4 (46–95)	< 0.001
Female (*n*)	38 (33.9%)	61 (47.2%)	0.003
Male (*n*)	74 (66.1%)	68 (52.8%)	0.343
BMI (mean, SD)	24.2 ± 4.1 (15–39)	25.9 ± 4.4 (17–39)	0.023
ASA score			
1–2 (*n*)	28 (21.4%)	52 (40.3%)	0.121
3–4 (*n*)	84 (78.6%)	77 (59.6%)	0.521
Vancouver			
B2 (*n*)	95 (15.2%)	91 (70.0%)	0.783
B3 (*n*)	17 (84.8%)	38 (30.0%)	0.058
Computed tomography required for diagnosis?			
Yes	22 (19.6%)	6 (4.7%)	
No	90 (80.4%)	123 (95.3%)	
Index surgery			
Osteoarthritis (*n*)	69	100	0.026
Femoral neck fracture (*n*)	32	22	0.085
Aseptic loosening of implant (*n*)	10	6	0.220
Other (*n*)	1	1	0.732
Initial implant			
Uncemented tapered stem (*n*)	25	69	0.012
Cemented tapered stem (*n*)	76	43	0.023
Cemented composite beam stem (*n*)	11	17	0.093
Years since index fracture (mean, SD)	5.5 ± 5.1 (0–16)	3.6 ± 6.1 (0–19)	0.322
Follow up in years (mean, SD)	3.9 ± 2.7 (0–12)	5.5 ± 3.9 (0–15)	0.182
Cognitive dysfunction			
No (*n*)	89 (79.5%)	119 (92.2%)	0.014
Possible/uncertain (*n*)	3 (2.6%)	3 (2.3%)	0.653
Definitive (*n*)	20 (17.1%)	7 (5.4%)	0.044

### Surgery

The stability of all stems was assessed preoperatively and classified as Vancouver B1 fractures if deemed stable and ineligible for the study or B2/B3 fractures with loose stem. Procedures were performed by 1 of 19 experienced consultant orthopedic surgeons specializing in traumatology or hip arthroplasty. The decision for implant revision or in situ fixation was at the discretion of individual surgeons. All surgeries utilized the posterior approach.

### Revision group

Stem revision procedures involved either an uncemented, distally anchored modular femoral stem (MP, Link Sweden, Stockholm) or a choice among cemented femoral stem options: CPT (Zimmer-Biomet, USA), Lubinus SP II (Waldemar Link, Hamburg, Germany), or the cemented, distally anchored modular femoral stem (MP, Waldemar Link, Hamburg, Germany). Cemented stems were categorized by length (short, < 150 mm and long, ≥ 150 mm). The approach for uncemented stems focused on meticulous cement removal from the femoral canal, while a “cement-in-cement” technique was employed for cemented stems, with minimal cement removal to accommodate the new stem [14, 15]. Supplementary stabilization techniques, such as cerclage wires or proximal femoral plates, were used as deemed clinically necessary.

### Outcomes and data collection

The outcomes encompassed hip-related complications, reoperations, and clinical outcomes. Data were retrieved using the Swedish personal identification number, hospital databases, routine follow-ups, and a digital case report form. The Swedish Arthroplasty Register aided in identifying reoperations beyond the Stockholm regional area.

### Variables

Data collection included patient demographics, cognitive status, ASA score, primary indication for surgery, time to radiological healing, and hip complications necessitating reoperations or closed reduction of dislocations. Fracture classification utilized the Vancouver B2–3 system, assessed by plain radiograph [16] and confirmed during surgery. Clinical and radiographic outcomes were evaluated through medical chart reviews and follow-up visits, grading patient outcomes through clinical evaluation on an arbitrary scale good, intermediate, poor, or deceased due to surgery. Clinical outcome was also based on fracture healing time. Radiological healing was assessed by radiologists, using plain radiographs, who provided reports stating that there was no evidence against healing or that healing was present. Clinical healing was evaluated by the treating physician; if there was radiological evidence that did not contradict healing, the patient was mobile, able to bear weight, had no severe pain, etc., it was considered healed.

### Statistics

No separate power calculation was performed for this descriptive study. A Cox proportional hazards regression model was utilized. The primary outcome was the time until reoperation or death. The covariates were BMI, age, sex, cognitive function, Vancouver class, ASA category, and surgical indication. Hazard ratios are presented as crude and adjusted hazard ratio. Descriptive statistics were used for groups comparisons. Between-group comparisons were conducted using Student’s ^t^-test, and a post-hoc Bonferroni correction was applied to the primary endpoint to manage multiplicity. Clinical score data, which were non-normally distributed and ordinal, respectively, underwent analysis using Mann–Whitney *U*-test. All statistical analyses were performed using SPSS 22.0.

### Ethics and registration

The study was conducted in accordance with the ethical principles of the Helsinki Declaration and was approved by the Ethics Committee of Karolinska Institutet (entry number dnr 2013/285-31/2).

## Results

Uncemented cases utilized mostly modular long stems while cemented cases had a mix of long stems, standard length stems and a few modular long stems. Some cases in both the cemented and uncemented groups also underwent simultaneous cup revision (Table 2).

**Table 2:** Treatment methods in the cemented and uncemented groups

	Cemented (*n* = 112)	Uncemented (*n* = 129)	*P* value
Stem used (*n*)			
Modular long uncemented stem		126 (97.7%)	N/A
Normal uncemented stem		3 (2.3%)	N/A
Long cemented stem	56 (50.0%)		N/A
Standard cemented stem	48 (42.8%)		N/A
Modular long cemented stem	8 (7.2%)		N/A
Stimultaneus plate fixation			
Yes	42 (37.5%)	4 (3.1%)	< 0.001
No	70 (62.5%)	125 (96.9%)	0.009
Stimultaneus cup revision			
Yes	14 (12.5%)	24 (18.6%)	0.034
No	98 (87.5%)	105 (81.4%)	0.291
Cup revision using dual mobility cup			
Yes	6 (42.8%)	3 (12.5%)	0.085
No	8 (57.2%)	21 (87.5%)	0.053

### Hip-related complications and reoperations

A total of 24 (12%) patients in the cemented group and 49 (38%) patients in the uncemented group were reoperated. Uncemented arthroplasty showed a significantly higher risk for reoperations compared with cemented arthroplasty when adjusted for variables [adjusted hazard ratio (HR) of 2.4, crude HR of 2.1]. Other factors such as sex, ASA category, Vancouver type, BMI, cognitive dysfunction, and surgical indication did not exhibit significant associations with reoperations (Table 3).

**Table 3:** Cox proportional hazard regression with crude and adjusted models

Variable	Years	PFF reoperations n (%)	n (%)	Adjusted HR	95% CI	Crude HR	95% CI
Age at revision surgery. mean (SD)	79 (9)			1.0	0.9 to 1.0	1.0	0.9 to 1.0
Treatment							
Cemented arthroplasty		23 (20)		1.0 ref		1.0 ref	
Uncemented arthroplasty		67 (38)		2.4	1.4 to 4.4	2.1	1.2 to 3.8
Sex							
Male			142 (59)	1.0 ref		1.0 ref	
Female			99 (41)	1.6	0.7 to 2.7	0.7	0.4 to 1.2
ASA category							
1–2			80 (33)	1.0 ref		1.0 ref	
3–4			161 (67)	1.6	0.3 to 4.0	1.3	0.4 to 2.9
BMI. mean (SD)			25.1 (4)	1.0	0.9 to 1.1	1.0	0.9 to 1.1
Cognitive dysfunction?							
No			208 (86)	1.0 ref		1.0 ref	
Possible/uncertain			6 (2)	0.5	0.4 to 2.3	0.4	0.1 to 2.9
Definitive			27 (11)	0.9	0.3 to 2.0	0.6	0.2 to 1.7
Indication for surgery							
Femoral neck fracture			55 (21)	1.0 ref		1.0 ref	
Osteoarthritis			168 (70)	0.7	0.3 to 1.3	0.9	0.5 to 1.7
Aseptic loosening of previous implant			16 (7)	0.5	0.1 to 1.7	0.7	0.2 to 2.0
Congenital Dysplasia of the hip			2 (1)	1.6	0.1 to 9.1	1.9	0.1 to 9.4
Vancouver type							
B2			186 (77)	1.0 ref		1.0 ref	
B3			55 (23)	1.1	0.5 to 2.4	1.0	0.7 to 1.5

Association with reoperations presented as hazard ratio (HR)

Significantly higher rates reoperation was observed in the uncemented group compared with the cemented group (*P* =  < 0.001). Cup revision and stem revision were the most common reoperations in the uncemented group, and debridement, antibiotics and implant retention (DAIR) was the most common reoperation in the cemented group (Table 4).

**Table 4:** Complications and reoperations between groups

Complication	Cemented (*n* = 112)	Uncemented (*n* = 129)	*P* value
Dislocation	10 (8.9%)	29 (22.5%)	0.004
PJI	8 (7.0%)	17 (13.1%)	0.124
Nonunion middle	1 (0.6%)	2 (1.6%)	0.648
Nonunion of trochanter	2 (1.5%)	6 (4.8%)	0.053
New PFX distal to stem (Vancouver C)	5 (4.3%)	2 (1.6%)	0.183
Loosening of stem	1 (0.6%)	12 (9.3%)	0.004
Number of complications	27	68	
Number of patients	24	49	
Revision surgery			
Cup revision	3 (2.3%)	16 (12.4%)	0.005
DAIR	8 (7.0%)	13 (10.0%)	0.424
Stem revision	2 (1.6%)	16 (12.4%)	0.003
Nonunion surgery	1 (0.6%)	1 (0.1%)	0.922
Change of proximal part of stem	0 (0%)	6 (4.7%)	0.022
Closed reduction	4 (3.4%)	9 (6.9%)	0.244
Distal plate fixation	5 (4.3%)	2 (1.6%)	0.182
Other	0 (0%)	4 (3.1%)	0.061
Number of surgeries	23	67	0.001
Reoperation rate	20.5%	37.9%	< 0.001

In the cemented group, the incidence of dislocation was lower (8.9%) compared with the uncemented group (22.5%) (*P* = 0.0043). No significant differences were found in the rates of periprosthetic joint infection (PJI), nonunion at various sites, new fractures distal to the stem (Vancouver C), or nonunion in the middle or trochanter. The uncemented group exhibited a higher incidence of stem loosening (9.3%) compared with the cemented group (0.6%) (*P* = 0.004). The total number of complications was 27 among 24 patients in the cemented group and 68 among 49 patients in the uncemented group (Table 4).

### Clinical outcome

The duration for fractures to heal was notably shorter in the cemented treatment group, averaging 11.4 weeks (±4.3), in contrast to 16.7 weeks (±8.8) in the uncemented group (*P* = 0.034). There were no statistical difference in clinical hip outcomes during follow-up. The time to death was notably shorter in the cemented group (47 weeks) compared with the uncemented group (Table 5).

**Table 5:** Clinical outcome between cemented and uncemented stem revision

Follow-up (mean)	Cemented (*n* = 112)	Uncemented (*n* = 129)	*P* value
Time to heal (weeks)	11.4 ± 4.3	16.7 ± 8.8	0.034
Hip outcome (mean)			
Good	72 (64.3%)	67 (51.9%)	0.362
Intermediate	24 (21.4%)	44 (34.1%)	0.063
Poor	8 (7.1%)	11 (8.5%)	0.194
Deceased	8 (7.1%)	4 (3.1%)	0.112
Dead	75 (68.7%)	71 (55.0%)	0.024
Time to death (months)	47 ± 13.6	64 ± 24.2	0.003

**Fig. 2:**
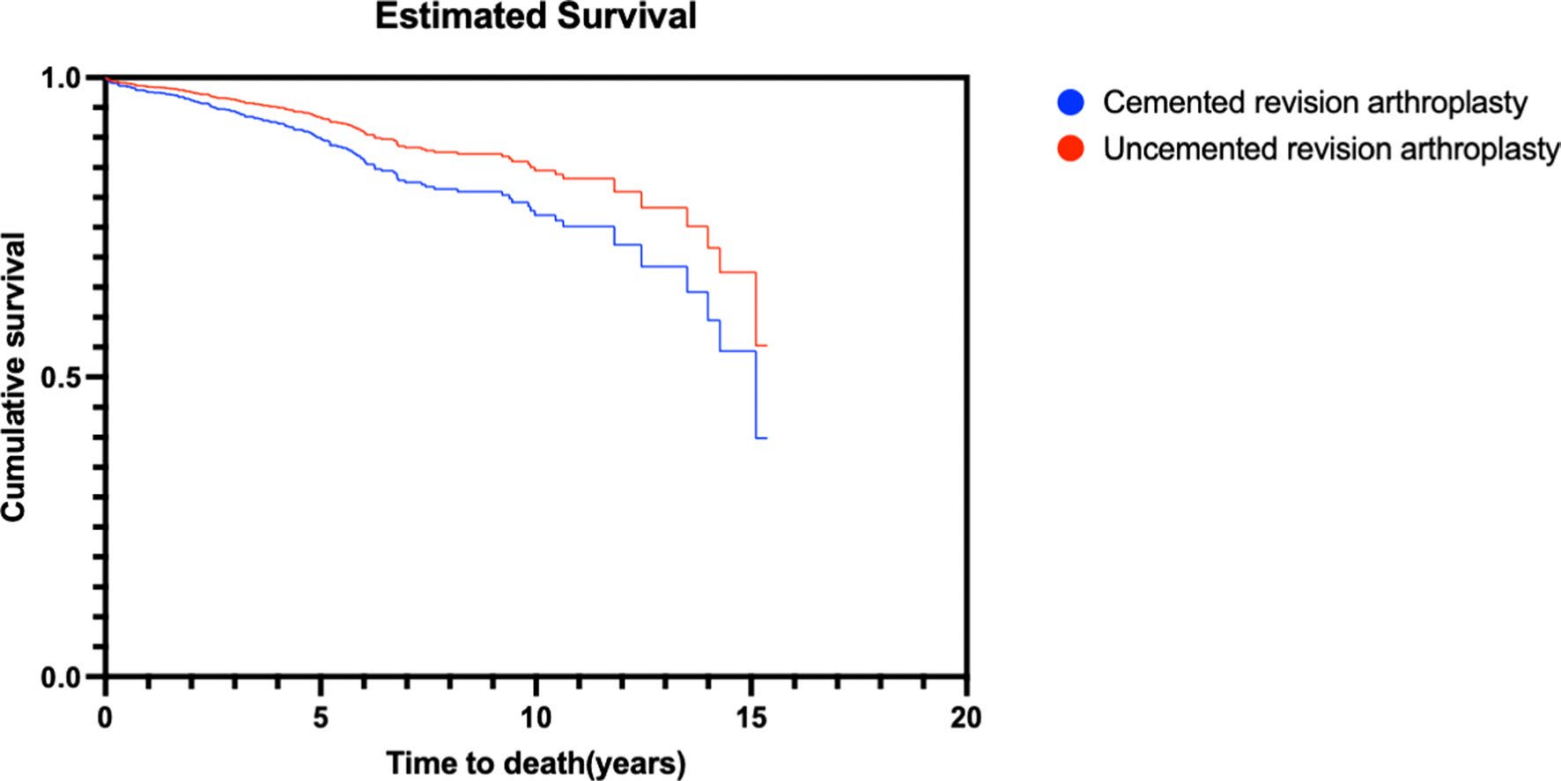
Cox regression of probability of death following revision surgery for PFF. Adjusted for BMI, age, sex, index surgery, ASA class, Vancouver type, and cognitive function

Mortality was higher in the cemented group compared with the uncemented group when adjusted for variables (*P* = 0.002) (Table 6) (Fig. 2).

**Table 6:** The 30 day, 1 year, and 2 year mortality rate

Mortality	Cemented (*n* = 112) %	Uncemented (*n* = 129) %	*P* value
30 days	7.1	1.6	0.008
1 year	15.2	11.6	0.013
2 years	23.2	16.3	0.002

Adjusted for BMI, age, sex, index surgery, ASA class, Vancouver type, and cognitive function

## Discussion

The management of PFF following hip arthroplasty, particularly the Vancouver B2 and B3 fractures associated with a loose femoral stem, is a challenge. In this retrospective cohort study, we found differences in complication and reoperation rate between the uncemented and cemented group. Patients with uncemented stems experienced almost double the complication rate requiring reoperations compared with cemented stems. Dislocations were the most common complications among both cemented and uncemented stems, which is in concordance with previous studies [17, 18]. Additionally, uncemented stems exhibited higher rates of stem loosening, the cause of this remains unanswered in this study. However, the higher rates of stem loosening among uncemented stems might be due to selection bias, as patients with cemented stems were older and might have died before experiencing stem loosening. Despite our findings of trends favoring better outcomes with cemented stems, we observed notably higher mortality rates in this group, accompanied by a shorter time to death among deceased patients. It is worth nothing that patients in the cemented group were both older and less cognitively intact than the uncemented group, possibly indicating a systematic selection bias and confounding. Surgeons might be more prone to choose cemented stem revision when dealing with frail and patient with comorbidities, skewing mortality data. Such an approach have been suggested by previous studies [19, 20].

The current scientific evidence is conflicting regarding the use of cemented versus uncemented stem in PFF management [17, 21–23]. While some studies suggest no definitive influence of fixation choice on implant survival, others indicate potential differences in implant survival rates, especially in older patients [24]. One study involving 86 patients with comparable femoral bone defects found no definitive influence of fixation choice on implant survival [25]. In contrast, another study of 209 patients indicated that uncemented revision stems led to inferior implant survival compared with cemented revision stems [26]. Furthermore, research based on registries suggests that uncemented revision stems might exhibit lower implant survival rates compared with cemented stems, especially in older patients [27–30]. These findings are shared by our study, which showed higher complication rates in uncemented stems. The use of cemented versus uncemented has also been shown to not compromise the healing of femoral fractures in elderly patients with osteoporotic bone, altered mobility, poor balance, and reduced cognitive capacity, this is in contrast to our findings, which indicate that cemented stem revision has a shorter time to healing than uncemented [20]. An alternative to stem revision might be ORIF, which is associated with lower blood loss, shorter operating times, and fewer reoperations [31–34].

The higher risk for revision surgery with uncemented stems due to dislocation is in line with register-based reports on elective revision surgery for stem loosening [8, 35]. The increased incidence of dislocation in uncemented revision stems could in part be the result of stem subsidence. The use of dual-mobility cups as part of the revision surgery could possibly reduce the rate of dislocation [36, 37].

Future prospective studies with more standardized protocols and randomization strategies could help mitigate biases and provide more robust evidence for guiding clinical decision-making in treating periprosthetic femoral fractures. Collaborative multicenter research can offer larger sample sizes and diverse populations for more robust analyses, in particular in terms of different stems used on cemented or uncemented revision. Understanding surgeon preferences and exploring alternative fixation methods, such as three-dimensional printed implants, are also promising areas for further investigation [38].

Surgeon preferences, clinical judgment, and individual patient characteristics might influence the selection of the surgical approach, leading to inherent differences between the groups observed in our study. The tendency to opt for a uncemented approach in Vancouver B3 fractures, driven by concerns about stability and the risk of complications, might influence the observed outcomes favorably for the uncemented group by selecting cases with better prognoses. In particular, fractures that are immediately anatomically reduceable might benefit from cemented approach [14]. While efforts were made to ensure comprehensive data collection and minimize biases through stringent inclusion and exclusion criteria, the retrospective nature of the study inherently limits our ability to account for these potential confounders and biases. Moreover, the Vancouver classification system is deemed reliable and effective in guiding surgeons when addressing periprosthetic femoral fractures; yet, its application can pose challenges, particularly for fractures surrounding cemented polished tapered stems [16, 39]. Proposed modifications to the classification system, such as introducing subclasses for intact cement-bone interfaces (B2W) and loose cement (B2L), aim to refine its applicability in such contexts [40].

This retrospective study highlights advantages in the cemented approach in managing PFF following hip arthroplasty, showing lower rates of dislocation, stem loosening, and shorter fracture healing times. However, caution is warranted in interpreting these findings due to inherent limitations, such as selection bias and higher mortality, in the cemented group. Further prospective research with standardized protocols is essential to refine treatment strategies and optimize patient care for periprosthetic femoral fractures.

## Data Availability

No additional data are available. Data are available on reasonable request to the corresponding author.

## Abbreviations

BMI: Body mass index
ORIF: Open reduction and internal fixation
PFF: Periprosthetic femoral fractures
PJI: Periprosthetic joint infection
REDCap: Research electronic data capture
STROBE: Strengthening the Reporting of Observational Studies in Epidemiology
